# Biobanks as fiduciaries of biosamples and data: a legal and ethical analysis in the light of international standards

**DOI:** 10.1093/jlb/lsag020

**Published:** 2026-07-07

**Authors:** Tatsuaki Tsuruyama, Takuya Hiratsuka

**Affiliations:** Department of Drug Discovery Medicine, Graduate School of Medicine, Kyoto University, Shogoin-kawahara-cho 53, Sakyo-ku, Kyoto 606-8507, Japan; Department of Drug Discovery Medicine, Graduate School of Medicine, Kyoto University, Shogoin-kawahara-cho 53, Sakyo-ku, Kyoto 606-8507, Japan

**Keywords:** biobank, fiduciary law, GDPR, ISO 20387, ISBER best practices, stewardship

## Abstract

This article argues that biobanks are best conceptualized as fiduciaries of biospecimens and associated data. Building on fiduciary legal theory and Anglo-American fiduciary jurisprudence, it explains how long-term discretionary control, informational asymmetry, and donors’ limited ability to monitor downstream uses justify duties of care and loyalty, even though legal recognition and enforceability remain jurisdiction-dependent. The argument is situated within earlier trust-based and fiduciary accounts of biobank governance and is connected to international governance standards, including ISO 20387 (which frames the biobank as a legal entity) and the International Society for Biological and Environmental Repositories Best Practices, treating them as practical specifications of fiduciary stewardship rather than sources of binding law. The fiduciary framework reconceives broad consent as an ongoing governance commitment rather than a one-time authorization, justifies cost recovery as reimbursement for professional stewardship, and subjects intellectual property and benefit-allocation arrangements to loyalty-based constraints. It also situates this fiduciary approach alongside EU General Data Protection Regulation principles of accountability, purpose limitation, and transparency. It argues that public trust is a condition of sustainable biobank governance and that fiduciary governance supports proactive, accountable promotion of research uses of samples and data.

## I. INTRODUCTION

Biobanks have become indispensable infrastructure for contemporary biomedical research, public health, and precision medicine. Large-scale population biobanks, disease-oriented repositories, and hospital-based biobanks now support longitudinal cohort studies, genomic medicine, drug discovery, and the secondary use of clinical data. The success of initiatives such as the UK Biobank has further accelerated the institutionalization of biobanking within national research strategies and global scientific collaboration.^1^^,^^2^ Despite this expansion and increasing social reliance, the legal characterization of biobanks remains underdeveloped and fragmented.

In many jurisdictions, biobanks are regulated primarily through a combination of research ethics review, data protection law, and sector-specific statutes. These frameworks typically focus on consent procedures, privacy safeguards, and institutional authorization.^3^ While such measures are indispensable, they do not adequately address a more fundamental question: what kind of legal and ethical relationship exists between biobanks and those who provide biosamples and associated data? The absence of a clear answer to this question has contributed to persistent uncertainty surrounding secondary use of samples, justification of cost recovery, allocation of intellectual property (IP), and the long-term governance of stored materials.^4^

This uncertainty is not merely theoretical. Donors often provide biosamples under conditions of broad or future-oriented consent, without knowing the precise scope of downstream research.^5^ Once samples and data are deposited, donors typically lack any practical ability to monitor how their contributions are stored, accessed, or reused. At the same time, biobanks exercise significant discretion over collection protocols, quality management, access decisions, and data linkage. These features create a structural asymmetry of information, expertise, and power that cannot be fully captured by traditional contractual or administrative models.^6^

The publication of ISO 20387 in 2018 marked a turning point in international biobanking governance. For the first time, an international standard explicitly defined a biobank as a ‘legal entity or part of a legal entity that performs biobanking’.^7^ This definition reflects an important normative shift: biobanks are no longer understood merely as technical storage facilities but as institutions bearing responsibility for complex socio-technical processes involving human biological materials and sensitive data. In parallel, the International Society for Biological and Environmental Repositories (ISBER) has updated its Best Practices for Repositories, most recently issuing the fifth edition, which articulates detailed expectations regarding infrastructure, quality management, personnel competence, data security, sustainability, and ethical oversight.^8^

Taken together, these international standards invite renewed examination of the legal status of biobanks. They also raise a critical normative question: if biobanks are legal entities entrusted with human biospecimens and data under conditions of legal uncertainty and donor vulnerability, what legal framework best captures their obligations toward donors and society?

Prior scholarship has already explored fiduciary and trust-based approaches to biobank governance. Within this trajectory, Winickoff and colleagues articulated a progression from a charitable trust model for genomic biobanks, to the ‘Biotrust’ as a social-contract framing of governance, and to analyses of UK Biobank as a ‘third way’ emphasizing institutional trust and access governance rather than ownership.^9^^,^^10^^,^^11^ More recently, related OECD work has examined how collaborative platforms in genomics and biobanks can be built and sustained through governance conditions that enable responsible sharing and use.^12^

In particular, earlier ‘biotrust’ proposals and related work conceptualized biobanks through the institutional structure of trust law, often using charitable trust analogies to frame stewardship obligations toward specimen contributors and the public. More recent debates in data governance, especially discussions of ‘information fiduciaries’ and critiques of that concept, have further highlighted both the appeal and the limits of fiduciary framing in contexts characterized by informational asymmetry, institutional discretion, and public dependence.

This article builds on, but differs from, those earlier approaches in two respects. First, it does not propose that biobanks must be constituted as formal trusts. Rather, it argues that biobank governance is usefully analyzed in fiduciary terms because biobanks exercise discretionary authority over entrusted biospecimens and associated data under conditions of asymmetry and limited donor monitoring. Second, it emphasizes that fiduciary framing does not merely constrain misconduct; it also clarifies the normative basis for proactive stewardship, including the responsible facilitation of research access, collaboration, and beneficial uses consistent with donor expectations and the public good.

This article argues that biobanks are most fruitfully conceptualized as fiduciary of biospecimens and associated data. Under this framework, biobanks owe duties of care and loyalty to donors and, in certain contexts, to the public at large. Fiduciary theory provides a unified account of why biobanks must prioritize stewardship over commodification, why informed consent must be interpreted dynamically rather than transactionally, and why transparency and sustainability are not optional ethical aspirations but legally relevant obligations.^13^

## II. INTERNATIONAL STANDARDS AND THE NORMATIVE STRUCTURE OF BIOBANKING

The argument in this article does not treat ISO 20387 or the ISBER Best Practices as sources of binding law. Rather, these instruments are analyzed as professional governance standards that help specify the practical content of institutional duties in biobanking, especially in relation to competence, quality management, traceability, and accountability. This professional-standard function is distinct from, but complementary to, broader ethical and policy frameworks developed by bodies such as the Organisation for Economic Co-operation and Development (OECD), World Health Organization (WHO), United Nations Educational, Scientific and Cultural Organization (UNESCO), and Global Alliance for Genomics and Health (GA4GH), which articulate higher-level governance principles while leaving many operational requirements under-specified.^14^

International biobanking standards are often described as technical instruments, yet their normative implications extend well beyond laboratory practice. ISO 20387 defines biobanking as a set of interrelated activities that include the collection, preparation, preservation, testing, analysis, and distribution of biological materials together with associated data.^15^ Crucially, the standard situates these activities within a legal entity that bears responsibility for governance, quality, and accountability across the entire lifecycle of biospecimens.

ISO 20387 establishes requirements for organizational governance, personnel competence, infrastructure, risk management, and quality systems. This governance-oriented emphasis is consistent with accounts of fiduciary law as ‘fiduciary governance’, which focus on structuring and constraining discretionary authority through institutional design and accountability mechanisms.^16^ These requirements presuppose that biobanks exercise professional judgment and discretion in circumstances where failures can compromise scientific validity, individual interests, and public trust.^17^ For example, the standard mandates continuous monitoring of storage conditions, documentation of deviations, and corrective actions to maintain sample integrity. It also requires controlled access to sensitive data, traceability of sample use, and mechanisms to ensure compliance with applicable ethical and legal norms.

The ISBER Best Practices for Repositories (fifth edition) complement ISO 20387 by providing detailed operational guidance grounded in decades of repository experience.^18^ The fifth edition places particular emphasis on sustainability planning, governance structures, data integrity, and risk-based quality management. This shift toward governance structures and sustainability planning also aligns with governance-oriented fiduciary theory, which emphasizes structuring and constraining discretionary authority through clear responsibilities and accountability mechanisms.^19^ Although framed as professional guidance rather than binding law, ISBER Best Practices function as de facto normative benchmarks within the global biobanking community.^20^

Importantly, neither ISO 20387 nor the ISBER Best Practices are value neutral. From the perspective of governance-oriented fiduciary theory, these emphases on fairness/impartiality, integrity, documentation, and auditable procedures are not merely technical. They function as institutional controls designed to structure and constrain discretionary authority in settings where informational asymmetry makes continuous monitoring difficult. In particular, documented and reviewable decision-making for access and secondary use can be understood as prophylactic mechanisms that reduce the risk of opportunism, understood not only as overt wrongdoing but also as structurally likely forms of bias or preferential treatment, by making exercises of discretion transparent, accountable, and contestable. In this sense, ISO 20387 and the ISBER Best Practices operationalize fiduciary governance by translating duties of care and loyalty into concrete procedural requirements.^21^^,^^22^^,^^23^^,^^24^ Embedded within these standards are assumptions about responsibility, accountability, and trust. The emphasis on documentation, transparency, and continuous improvement reflects an expectation that biobanks must be able to justify their decisions to stakeholders who lack equivalent expertise or access to relevant information.^25^ Similarly, explicit requirements for sustainability planning acknowledge that biobanks assume long-term custodial responsibilities that extend beyond individual research projects or funding cycles.^26^

From the donor’s perspective, participation in a biobank is not a discrete transaction but an act of entrustment. Donors relinquish control over physical samples and data, often motivated by expectations that their contributions will be used responsibly to advance science or public health.^27^ International standards respond to this expectation indirectly by imposing professional obligations on biobanks, yet they stop short of articulating the underlying legal relationship that gives these obligations coherence.

A fiduciary framework provides a principled basis for interpreting and applying international biobanking standards. Viewed through a fiduciary lens, ISO 20387 and ISBER Best Practices can be understood as specifying the content of professional duties owed by biobanks. Infrastructure and quality requirements correspond to the duty of care; consent governance and access controls reflect the duty of loyalty; and sustainability planning addresses the obligation to preserve entrusted assets for their intended purposes.^28^^,^^29^ In this way, international standards supply the material foundation for fiduciary responsibility, while fiduciary theory supplies the legal and ethical rationale that explains why such standards are necessary and how they should be interpreted.

## III. FIDUCIARY THEORY AND LEGAL FOUNDATIONS

Fiduciary law provides a legal framework for analyzing relationships characterized by discretionary power, informational asymmetry, and donor vulnerability. Unlike contract law, which is organized around negotiated exchange, fiduciary doctrine is often invoked where one party exercises judgment over another’s important interests in circumstances that cannot be fully specified or monitored ex ante. Its core function is to impose heightened duties of conduct, most notably the duties of care and loyalty, on actors entrusted with such discretion.^30^

At the same time, the present argument distinguishes between a normative and a doctrinal claim. Normatively, the structure of the donor–biobank relationship provides strong reasons to analyze biobank governance in fiduciary terms. Doctrinally, however, legally enforceable fiduciary duties depend on recognition through jurisdiction-specific law, including judicial doctrine, legislation, regulation, or institutional governance arrangements. The argument advanced here is therefore not that all biobank relationships are already recognized as fiduciary in law, but that fiduciary theory offers the most coherent framework for specifying and evaluating biobank obligations.

Frankel’s influential account identifies fiduciary relationships as those in which one party undertakes to act for the benefit of another in matters involving discretion and trust.^31^ The fiduciary is granted latitude to make decisions, while the beneficiary remains dependent on the fiduciary’s expertise and integrity. This combination of discretion and dependency explains why fiduciary law departs from ordinary standards of arm’s-length exchange and instead imposes strict obligations designed to prevent abuse.

Recent fiduciary theory helps sharpen this analysis in at least two respects. A prominent-account emphasizes fiduciary law’s role in constraining opportunism, understood not only as overt misconduct but also as structurally likely forms of bias or preferential treatment, when an institution holds discretionary power over entrusted interests under conditions of informational asymmetry.^32^ First, governance-oriented accounts emphasize that fiduciary law is centrally concerned with the administration of entrusted interests under conditions of discretionary authority, not merely with isolated acts of trust or reliance.^33^ Second, more restrictive theoretical accounts caution against treating all asymmetric or trust-based relationships as fiduciary and instead stress the need to identify the institutional and relational features that justify fiduciary obligations in a given context.^34^ Taken together, these refinements support the present argument’s distinction between a normative fiduciary analysis of biobank governance and the separate question of jurisdiction-specific legal recognition and enforceability.^35^^,^^36^

Biobanks exhibit these structural features with clarity. Donors entrust biospecimens and associated data to institutions that hold themselves out as possessing specialized scientific, technical, and organizational expertise. Once samples are deposited, biobanks exercise wide discretion over storage conditions, quality control, access policies, data linkage, and the authorization of secondary uses. Donors, by contrast, typically lack the ability to oversee or meaningfully influence these decisions over time. This asymmetry is compounded by the long-term nature of biobanking, where materials may be retained and reused for decades beyond the initial context of collection. For these reasons, fiduciary theory provides a particularly useful framework for analyzing biobank obligations, even where positive law has not yet formally classified biobanks as fiduciaries.

Fiduciary theory thus offers a compelling lens through which to understand the normative position of biobanks. Rather than treating biobanks as mere service providers or custodians, fiduciary law recognizes them as institutions entrusted with interests that are morally and legally significant. These interests include not only the physical integrity of biospecimens but also the informational, privacy-related, and dignity-related concerns associated with genetic and health data.^37^ It is also important to distinguish trust-based governance models from fiduciary analysis more generally. A trust is a specific legal institution for the management of assets, whereas fiduciary duties are a broader category of obligations that may arise in a range of legal and institutional relationships involving entrusted discretion. Trustees are fiduciaries, but not all fiduciaries are trustees. Earlier proposals such as Winickoff and Winickoff’s charitable trust model and subsequent ‘Biotrust’ formulations illuminate an important trust-based path for biobank governance, but they do not exhaust the fiduciary question.^38^^,^^39^ The present article does not require biobanks to be constituted as formal trusts. Instead, it argues that biobanks, as institutions exercising long-term discretionary stewardship over biospecimens and associated data, can be evaluated and regulated through a fiduciary framework even where trust law is not adopted as the governing legal form. A related move in data-governance theory is the ‘information fiduciary’ proposal, which extends fiduciary reasoning to digital platforms handling personal data; however, that proposal has also attracted significant criticism regarding conceptual fit and practical scope. The present argument draws on this debate comparatively but is grounded in the distinct institutional features of biobank governance.^40^^,^^41^

## IV. DUTIES OF LOYALTY AND THE PROHIBITION OF UNAUTHORIZED BENEFIT

The duty of loyalty is a defining feature of fiduciary law. It requires fiduciaries to act in the interests of beneficiaries and to avoid conflicts between fiduciary responsibilities and personal or institutional gain. Classic jurisprudence articulates this duty in particularly stringent terms.

In Keech v. Sandford (1726), the English Court of Chancery held that a trustee who obtained a lease renewal for his own benefit, after the beneficiary had been denied renewal, had breached his fiduciary duty.^42^ The court emphasized that the trustee could not profit from an opportunity arising from the trust relationship, even if the beneficiary could not have obtained the benefit independently. This strict approach reflects a foundational principle of fiduciary law: loyalty is enforced prophylactically, not merely to remedy actual harm but to eliminate incentives for self-dealing.

The relevance of Keech v. Sandford to biobanking lies in its articulation of fiduciary loyalty as an obligation independent of outcome. Biobanks routinely face opportunities to derive institutional benefit from entrusted samples and data, whether through cost recovery schemes, collaborations with commercial entities, or facilitation of downstream IP. Under a fiduciary framework, such activities are not per se prohibited. However, they must be justified by reference to donor authorization, alignment with donor expectations, or demonstrable benefit to the entrusted purpose. Absent such justification, the appropriation of value derived from entrusted materials risks constituting a breach of loyalty.^43^

This principle has direct implications for debates over IP arising from biobank-mediated research. This does not imply that downstream IP rights are categorically incompatible with fiduciary biobank governance, nor that donors must hold formal IP rights in subsequent inventions. Rather, fiduciary loyalty requires transparent justification of how IP and related benefits are allocated, and scrutiny of arrangements that may undermine access to underlying biobank resources, distort entrusted purposes, or generate unaddressed conflicts of interest. The fiduciary question is therefore not simply who owns downstream IP, but whether the governance of IP remains consistent with loyal and accountable stewardship of entrusted materials and data.^44^

## V. INFORMATION ASYMMETRY AND THE DUTY OF CARE

The duty of care complements the duty of loyalty by requiring fiduciaries to exercise appropriate skill, diligence, and judgment in managing entrusted interests. Courts have consistently recognized that the content of this duty depends on the fiduciary’s claimed expertise and the context of the relationship.

In the USA, the Supreme Court articulated this principle in SEC v. Capital Gains Research Bureau, Inc. (1963). The Court held that investment advisers owe fiduciary duties to their clients due to the informational asymmetry inherent in the advisory relationship.^45^ Importantly, the Court emphasized that fiduciary obligations arise not from explicit contractual terms but from the adviser’s position of trust and the client’s reliance on professional judgment.

Biobanks occupy an analogous position. By presenting themselves as professionally managed repositories compliant with international standards, biobanks invite reliance on their expertise in areas such as sample preservation, data security, and regulatory compliance. The duty of care thus requires biobanks to maintain infrastructure, quality management systems, and operational procedures that meet or exceed accepted professional norms.^46^^,^^47^ Failures in storage conditions, data governance, or sustainability planning are not merely technical lapses but potential breaches of fiduciary care.

ISO 20387 and the ISBER Best Practices can be understood as articulating the expected content of this duty. Requirements for monitoring storage conditions, validating information systems, and ensuring personnel competence specify what reasonable care entails in the biobanking context. Under a fiduciary interpretation, compliance with these standards is not optional or purely reputational; it forms part of the legal and ethical obligation owed to those who have entrusted their biological materials.^48^^,^^49^

## VI. WHY CONTRACTUAL MODELS ARE INSUFFICIENT

Some commentators have suggested that the relationship between donors and biobanks can be adequately described through contract law, with informed consent functioning as the primary contractual mechanism. This view, however, fails to account for several structural features of biobanking.

First, informed consent in biobanking rarely resembles negotiated agreement. Consent documents are typically standardized, offered on a take-it-or-leave-it basis, and oriented toward future, unspecified research uses. Donors do not bargain over terms, nor do they possess the information necessary to assess the full range of potential downstream uses.^50^ Second, contract law presumes relatively symmetrical information and the ability to monitor performance, conditions that are plainly absent in long-term biobank participation.

Third, contractual models struggle to accommodate the open-ended temporality of biobanking. Contracts are generally designed to govern discrete transactions or defined periods, whereas biobanks assume custodial responsibilities that may extend indefinitely. Fiduciary law, by contrast, is explicitly designed to regulate relationships of ongoing discretion and trust.^51^

More fundamentally, informed consent in biobanking should not be treated as a contract or as a one-time settlement of governance responsibilities. It is typically a revocable authorization grounded in voluntariness, rather than a bilateral exchange of binding obligations. Donors generally retain the possibility of withdrawal from further participation or future uses of their materials or data, where feasible, subject to practical limits, for example, where materials or data have already been anonymized, distributed, or incorporated into research outputs. This revocable and non-contractual character reinforces the point that consent forms do not, by themselves, define or exhaust the governance responsibilities of biobanks.

## VII. LIMITATIONS OF PUBLIC LAW AND ADMINISTRATIVE MODELS

Public law and administrative oversight play an important role in biobank governance, particularly in ensuring compliance with data protection and research ethics requirements. However, public law frameworks tend to focus on institutional authorization and procedural safeguards rather than individualized duties owed to donors.

While ethics committees and regulatory authorities can impose baseline standards, they cannot fully substitute for relational obligations grounded in trust. Fiduciary theory complements public law by explaining why biobanks must act impartially and responsibly even in areas where explicit regulation is silent or permissive.^52^ It thus provides a normative bridge between institutional oversight and donor-centered obligations.

## VIII. FIDUCIARY THEORY AS AN INTEGRATIVE FRAMEWORK

Taken together, fiduciary duties of care and loyalty offer an integrative framework for understanding biobank governance. They explain why biobanks must prioritize stewardship over commodification, why conflicts of interest must be carefully managed, and why long-term sustainability is an ethical and legal necessity rather than a managerial preference.

By grounding these obligations in fiduciary legal theory, the framework developed here helps translate biobank ethics into standards of conduct that can be institutionalized, and, in some settings, made enforceable, through legal, regulatory, professional, and organizational governance mechanisms. In this sense, fiduciary theory does not presuppose that courts already recognize biobanks as fiduciaries in all jurisdictions; rather, it provides a conceptual architecture for designing and evaluating governance arrangements that give concrete effect to duties of care, loyalty, accountability, and stewardship.

The enforceability of fiduciary standards in biobanking may arise through multiple, complementary pathways. These include statutory or regulatory recognition of specific duties, incorporation of fiduciary principles into oversight and accreditation frameworks, and institutional governance mechanisms such as independent review, conflict-of-interest controls, auditability, and transparency requirements. Professional standards (including those reflected in ISO and ISBER-based practices) may also function as benchmarks for assessing whether biobanks have exercised reasonable care and loyal stewardship. The point is not that a single legal route is universally available, but that fiduciary theory can guide the design of governance systems in which biobank obligations become more concrete, reviewable, and sanctionable. This understanding is consistent with recent Japanese biobank initiatives that frame biobanks not merely as repositories but as research infrastructures for facilitating the use of samples and associated data. For example, a review of the National Center Biobank Network (NCBN) reports that, since FY2018, NCBN has participated in the AMED-led B-Cure ‘biobank cross-search system’, which promotes the utilization of bioresources among Japan’s three major biobanks (NCBN, BioBank Japan, and the Tohoku Medical Megabank Project.^53^^,^^54^^,^^55^ Concrete pathways include (i) accreditation and audit mechanisms linked to professional standards (eg, ISO-based biobank accreditation) and (ii) administrative enforcement under data protection regimes [eg, supervisory authority oversight and penalties under general data protection regulation (GDPR)], which can make governance failures practically sanctionable beyond private litigation.^56^^,^^57^

## IX. APPLICATIONS: CONSENT, COST RECOVERY, INTELLECTUAL PROPERTY, AND DATA GOVERNANCE

The fiduciary framework developed above has direct implications for some of the most contested issues in contemporary biobank governance: informed consent, cost recovery, IP, and data governance. These issues are often treated as separate regulatory or managerial problems. However, when viewed through the lens of fiduciary law, they can be understood as interconnected manifestations of the duties of care and loyalty owed by biobanks to donors and, in certain contexts, to the public.^58^^,^^59^^,^^60^ An important implication of the fiduciary framework is that biobanks are not merely passive repositories. Fiduciary stewardship entails a degree of discretionary responsibility to promote the legitimate and beneficial use of entrusted biospecimens and associated data. In practice, this may include facilitating ethically and scientifically appropriate access, coordinating collaborations, improving discoverability and quality of collections, and supporting research uses that are consistent with donor expectations and the public good. Thus, fiduciary governance is not only a framework of restraint; it also provides a normative basis for proactive, accountable research facilitation.^61^ This emphasis on responsible facilitation and access governance echoes prior analyses of UK Biobank as a governance ‘third way’, as well as policy work on sustaining collaborative genomics/biobank platforms through institutional design and trust-enabling governance conditions.^62^^,^^63^

## X. INFORMED CONSENT AS AN ONGOING FIDUCIARY PROCESS

In biobank practice, informed consent is frequently conceptualized as a procedural requirement satisfied at the point of sample collection. Consent forms typically authorize broad or future research uses, reflecting the inherent uncertainty of scientific development. While such consent models are widely accepted, they have also generated ethical concern regarding donor autonomy and informational adequacy.^64^

A fiduciary perspective reframes informed consent not as a discrete contractual authorization but as part of an ongoing relationship of trust. Fiduciary law recognizes that consent obtained at one point in time cannot exhaustively authorize future exercises of discretion where circumstances, risks, or uses materially change. Instead, fiduciary duties require continuous attentiveness to the interests and reasonable expectations of beneficiaries.^65^

Applied to biobanking, this means that initial consent does not absolve biobanks of responsibility for downstream governance decisions. Decisions regarding secondary use, data linkage, or collaboration with commercial partners must be evaluated in light of donor expectations, evolving social norms, and the original purpose of entrustment.^66^ Where uses deviate substantially from what donors could reasonably have anticipated, fiduciary loyalty may require additional governance mechanisms, such as re-consent, enhanced oversight, or public justification.

This interpretation aligns with international standards, which emphasize accountability and transparency throughout the biobanking lifecycle.^67^^,^^68^ It also provides a principled explanation for why consent in biobanking cannot be reduced to a one-time waiver of rights, even where broad consent is formally valid.

## XI. COST RECOVERY AND THE PROHIBITION OF COMMODIFICATION

Another persistent source of controversy concerns the legitimacy of charging fees for access to biospecimens and data. Critics have argued that such fees risk commodifying human biological materials, undermining respect for persons and donor trust.^69^ Biobanks, on the other hand, face substantial financial burdens associated with infrastructure maintenance, quality management, and long-term sustainability.

Fiduciary theory offers a coherent resolution to this tension. Under fiduciary law, compensation for services is permissible where it reflects the exercise of professional skill and care in managing entrusted assets. What fiduciary law prohibits is the extraction of profit derived from self-dealing or the exploitation of entrusted property for purposes unrelated to the beneficiary’s interests.^70^

From this perspective, cost recovery in biobanking is best understood as an administrative and professional mechanism for sustaining responsible stewardship, not as a claim of proprietary entitlement over biospecimens themselves. The core fiduciary concern is therefore not the existence of fees as such, but whether cost structures are transparent, proportionate, and consistent with the entrusted purposes of the biobank.^71^^,^^72^ By contrast, pricing models that treat samples as market commodities, valued according to scarcity or demand, risk violating fiduciary loyalty by subordinating donor interests to institutional or commercial gain.

International best practices implicitly endorse this distinction. Both ISO 20387 and the ISBER Best Practices stress the importance of transparent cost accounting and sustainability planning, without endorsing profit-maximizing models.^73^^,^^74^ Fiduciary theory explains why such restraint is normatively required: the entrusted nature of biospecimens constrains how value derived from them may be legitimately realized.

## XII. INTELLECTUAL PROPERTY AND FIDUCIARY LOYALTY

Questions of IP arising from biobank-mediated research further illustrate the analytical power of fiduciary theory. Disputes over IP are often framed as matters of ownership or contractual allocation between institutions and users. However, this framing obscures the fiduciary dimension of biobanking.

Where IP arises from research conducted on entrusted biospecimens and data, fiduciary loyalty requires careful scrutiny of how benefits are allocated. Fiduciary law places the burden on the fiduciary to demonstrate that any appropriation of benefits is consistent with the beneficiary’s interests or has been explicitly authorized.^75^^,^^76^ In the absence of clear donor consent or benefit-sharing arrangements, the unilateral attribution of IP to researchers or commercial entities may conflict with fiduciary obligations.

This does not imply that donors must hold formal IP rights in downstream inventions. Rather, fiduciary theory requires that governance frameworks transparently address how benefits are distributed and ensure that arrangements do not undermine the purpose for which samples were entrusted.^77^^,^^78^ This concern is underscored by governance debates in African genomics and biobanking, which emphasize community engagement, benefit sharing, and capacity building as conditions of legitimate access and reuse.^79^^,^^80^ This fiduciary approach is particularly relevant in contexts involving vulnerable populations or indigenous communities, where historical patterns of exploitation have eroded trust in research institutions.^81^

## XIII. DATA GOVERNANCE, GDPR, AND FIDUCIARY RESPONSIBILITY

The fiduciary framework also illuminates the relationship between biobanking and data protection law, particularly under the European Union’s GDPR.^82^ GDPR is premised on protecting data subjects in contexts of personal-data processing marked by informational asymmetry, including long-term data processing for scientific research.^83^ Although GDPR does not explicitly invoke fiduciary terminology, its core principles closely parallel fiduciary duties of care and loyalty.

Principles such as lawfulness, purpose limitation, data minimization, and accountability reflect expectations that data controllers exercise discretion responsibly and in alignment with the interests of data subjects.^84^^,^^85^ When biobanks process sensitive health and genetic data, they occupy a position analogous to fiduciaries: entrusted with information that individuals cannot realistically monitor or control once disclosed.

Conceptualizing biobanks as fiduciaries provides a normative explanation for why GDPR imposes heightened obligations on data controllers in research contexts. It also clarifies why compliance with GDPR should be understood as a baseline rather than a ceiling. Fiduciary responsibility extends beyond formal legal compliance to encompass broader obligations of transparency, justification, and trustworthiness.^86^^,^^87^

The comparison to fiduciary obligations is therefore functional and normative rather than doctrinal: GDPR does not designate biobanks as fiduciaries, but its principles illuminate regulatory concerns that fiduciary theory helps to organize and interpret.

## XIV. PUBLIC TRUST AND THE COLLECTIVE DIMENSION OF FIDUCIARY GOVERNANCE

Biobanks often serve public or quasi-public purposes, supporting population health research and policy development. In such contexts, fiduciary obligations may extend beyond individual donor relationships to encompass duties toward the public as a collective beneficiary.^88^

Public trust is not merely an instrumental resource for recruitment but a normative condition for the legitimacy of biobanking. Fiduciary governance helps explain why biobanks must act impartially, manage conflicts of interest, and maintain openness regarding governance structures and decision-making processes. Practices such as public reporting, independent oversight, and stakeholder engagement can thus be understood as expressions of fiduciary loyalty at an institutional level.^89^

This collective dimension does not negate individualized duties to donors. Rather, fiduciary theory provides a framework for balancing individual and collective interests in a principled manner. By recognizing biobanks as fiduciaries, law and policy can better articulate the conditions under which trust is warranted and the circumstances in which institutional discretion must be constrained.

## XV. CONCLUSION AND POLICY IMPLICATIONS

This article has argued that biobanks are usefully analyzed as fiduciary of biospecimens and associated data. By situating biobank governance within fiduciary legal theory, it has shown that the core obligations in biobanking, care, loyalty, accountability, and stewardship, are not merely ad hoc ethical aspirations or technical requirements, but structurally grounded in relationships of entrusted discretion, informational asymmetry, and donor vulnerability. Whether and how these duties become legally enforceable depends on jurisdiction-specific legal and institutional arrangements.^90^^,^^91^

The fiduciary framework clarifies why existing regulatory approaches, taken in isolation, remain insufficient. Contractual models fail to account for the non-negotiated, long-term, and informationally asymmetric nature of donor participation. Public law and administrative oversight, while essential, focus primarily on procedural compliance and institutional authorization, leaving under-theorized the relational duties owed to individual donors. Fiduciary law, by contrast, is designed precisely to govern relationships in which one party exercises discretion over interests that cannot be fully specified or monitored ex ante.^92^^,^^93^^,^^94^

By integrating fiduciary theory with international standards such as ISO 20387 and the ISBER Best Practices (fifth edition), this article has demonstrated how technical governance requirements acquire normative coherence. Infrastructure maintenance, quality management systems, sustainability planning, and controlled access to samples and data are not merely best practices; they are expressions of fiduciary duties of care and loyalty.^95^^,^^96^^,^^97^ This perspective explains why biobanks must continuously justify their decisions to donors and stakeholders, even where formal legal compliance has been achieved.

The fiduciary framework also offers a unifying account of contentious practical issues. Informed consent emerges as an ongoing governance process rather than a one-time contractual waiver. Cost recovery is justified as compensation for professional stewardship, not as commodification of human biological materials. IP arrangements must be scrutinized for consistency with donor authorization and fiduciary loyalty, placing the burden of justification on biobanks rather than on donors.^98^^,^^99^^,^^100^^,^^101^ In each case, fiduciary law provides principled criteria for distinguishing legitimate institutional discretion from impermissible self-interest.

Importantly, conceptualizing biobanks as fiduciaries aligns closely with contemporary data protection regimes, particularly the EU GDPR. Although GDPR does not explicitly invoke fiduciary terminology, its emphasis on accountability, purpose limitation, and protection of vulnerable data subjects reflects concerns analogous to those addressed by fiduciary law.^102^^,^^103^ Fiduciary theory thus helps explain why GDPR imposes heightened obligations on biobanks handling sensitive health and genetic data and why mere formal compliance cannot exhaust biobank responsibility.^104^

Beyond individual donor relationships, fiduciary governance also illuminates the collective dimension of biobanking. Many biobanks operate as public or quasi-public institutions, contributing to population health research and policy development. In such contexts, fiduciary obligations extend to maintaining public trust as a shared good. Transparency, independent oversight, and stakeholder engagement are not optional add-ons but institutional manifestations of fiduciary loyalty at the collective level.^105^^,^^106^

From a policy perspective, recognizing biobanks as fiduciaries has several implications. Legislators and regulators may consider explicitly incorporating fiduciary language into biobank governance frameworks, thereby clarifying the nature of biobank obligations beyond procedural compliance. Ethics committees and oversight bodies can draw on fiduciary principles to evaluate governance practices that fall outside the scope of existing regulations. International standard-setting organizations may further articulate how fiduciary responsibilities inform the interpretation and implementation of technical requirements.

Finally, this analysis underscores the ethical significance of professional integrity in biobanking. Fiduciary law presupposes not only external regulation but also internal commitment to acting in the interests of others. In this sense, the fiduciary framework reinforces the moral foundations of biobanking while providing legal tools to address breaches of trust. As biobanking continues to expand in scope and societal importance, a fiduciary approach offers a coherent, durable, and ethically consistent foundation for governance. A further contribution of the fiduciary framework is to clarify the normative basis for proactive biobank stewardship. Biobanks are not only obligated to avoid misuse; they also bear responsibility for facilitating legitimate and socially valuable research uses of entrusted resources in ways that remain accountable to donors, institutions, and the public.

## Footnotes

1 L. Coppola et al., *Biobanking in Health Care: Evolution and Future Directions*, 17 J Transl  Med. 172 (2019).

2 L. Spinney, *UK Launches Tumor Bank to Match Maligned Biobank*, 9 Nat  Med. 491 (2003).

3 H. Langhof, J. Schwietering, & D. Strech, *Practice Evaluation of Biobank Ethics and Governance: Current Needs and Future Perspectives*, 56 J Med  Genet. 176–185 (2019).

4 J. W. Goebel et al., *Legal and Ethical Consequences of International Biobanking from a National Perspective: the German BMB-EUCoop Project* 18 Eur J Hum  Genet. 522–525 (2010).

5 L. Johnsson, Trust in  Biobank  Research: Meaning and  Moral  Significance (Uppsala University, 2013).

6 P. H. Watson, *Biobank Classification: Communicating Biorepository Diversity*, 12 Biopreserv  Biobank. 163–164 (2014).

7 International Organization for Standardization, ISO 20387:2018 Biotechnology—Biobanking—General Requirements for Biobanking.

8 ISBER, Best  Practices for  Repositories (5th ed. International Society for Biological and Environmental Repositories, 2023) (contributors: A. S. Abdelhafiz, R. Aguilar Quesada, C. Battiston, M. Bledsoe, M. Castelhano, D. Chadwick, N. Chakraborty, M. Edler, H. Ellis, S. Furlong, N. Gabancho, D. L. Garcia, L. Healy, M. Henderson, S. Higgins, P. Holanda, C. Hunt, R. Hurtado-Ortiz, E. James, P. Kilbride, M. Kulkarni, C. Lessard, K. Lloyd, M. Maseme, A. Michalska-Falkowska, C. Michels, A. Moors, H. Nansumba, T. Nguyen, A. Ovcharenko, S. Paul, J. P. Jeon, I. Pope, R. Pugh, D. Rainbolt, N. Ramirez, W. Schleif, O. Seberg, P. Singh, G. Stacey, T. Tsuruyama, R. Upton, R. Vollmer, C. Willers, E. Woods, and A. Zaayenga).

9 D. E. Winickoff & R. N. Winickoff, *The Charitable Trust as a Model for Genomic Biobanks*, 349 N Engl J Med. 1180–1184 (2003).

10 D. E. Winickoff & L. B. Neumann, *Toward A Social Contract for Genomics: Property and the Public in the ‘Biotrust’ Model*, 1 Genomics, Society and  Policy. 8–21 (2005).

11 D. E. Winickoff, *Partnership in U.K. Biobank: A Third Way for Genomic Property?*, 35 J Law  Med  Ethics. 440–456 (2007).

12 H. Garden, N. Hawkins & D. E. Winickoff, Building and Sustaining Collaborative Platforms in Genomics and Biobanks for Health Innovation (OECD Science, Technology and Industry Policy Papers, No. 102, 2021).

13 T. Frankel, *Fiduciary Law*, 71 Calif L Rev. 795–836 (1983).

14 Garden, Hawkins, & Winickoff, *supra* note 12.

15 International Organization for Standardization, *supra* note 7.

16 P. B. Miller & A. S. Gold, *Fiduciary Governance*, 57 William & Mary  Law  Review. 513–586 (2015).

17 International Organization for Standardization, *supra* note 7.

18 ISBER, *supra* note 8.

19 Miller & Gold, *supra* note 16.

20 L. D. Campbell et al., *The 2018 Revision of the ISBER Best Practices: Summary of Changes and the Editorial Team’s Development Process*, 16 Biopreserv  Biobank. 3–6 (2018).

21 International Organization for Standardization, *supra* note 7.

22 ISBER, *supra* note 8.

23 Miller & Gold, *supra* note 16.

24 D. G. Smith, *The Critical Resource Theory of Fiduciary Duty*, 55 Vanderbilt  Law  Review. 1397 (2002).

25 A. H. Virani & H. Longstaff, *Ethical Considerations in Biobanks: How a Public Health Ethics Perspective Sheds New Light on Old Controversies*, 24 J Genet  Couns. 428–432 (2015).

26 P. H. Watson et al., *A Framework for Biobank Sustainability*, 12 Biopreserv  Biobank. 60–68 (2014).

27 H. Yaghoobi & S. A. Hosseini, *History of the Largest Global Biobanks, Ethical Challenges, Registration, and Biological Samples Ownership*, 31 J Public  Health. 467–477 (2023).

28 Frankel, *supra* note 13.

29 Watson et al., *supra* note 26.

30 Frankel, *supra* note 13.

31
*Id*.

32 Smith, *supra* note 24.

33 Miller & Gold, *supra* note 16.

34 Smith, *supra* note 24.

35 Miller & Gold, *supra* note 16.

36 Smith, *supra* note 24.

37 Winickoff, *supra* note 11.

38 Winickoff & Winickoff, *supra* note 9.

39 Winickoff & Neumann, *supra* note 10.

40 J. M. Balkin, *Information Fiduciaries and the First Amendment*, 49 UC Davis  Law  Review. 1183–1234 (2016).

41 L. M. Khan & D. E. Pozen, *A Skeptical View of Information Fiduciaries*, 133 Harv L Rev. 497 (2019).

42 Keech v. Sandford (1726) 25 Eng. Rep. 223 (Ch.).

43 M. Jordan, J. Liddicoat & K. Liddell, *An Empirical Study of Large, Human Biobanks: Intellectual Property Policies and Financial Conditions for Access*, 8 J Law  Biosci. lsab018 (2021).

44 J. Simon & J. Robienski, *Property, Personality Rights and Data Protection with Regard to Biobanks*, 20 J Int  Bioethique. 47–56 (2009).

45 SEC v. Capital Gains Research Bureau, Inc., 375 U.S. 180 (1963).

46 International Organization for Standardization, *supra* note 7.

47 ISBER, *supra* note 8.

48 International Organization for Standardization, *supra* note 7.

49 ISBER, *supra* note 8.

50 Langhof, Schwietering & Strech, *supra* note 3.

51 Winickoff & Winickoff, *supra* note 9.

52 C. Porteri, E. Togni & P. Pasqualetti, *The Policies of Ethics Committees in the Management of Biobanks Used for Research: An Italian Survey,* 22 Eur J Hum  Genet. 260–265 (2014).

53 Y. Omae, Yu-Ichi Goto & K. Tokunaga, *National Center Biobank Network*, 9 Hum  Genome  Var. 38 (2022), https://doi.org/10.1038/s41439-022-00217-6.

54 A. Nagai et al., *Overview of the BioBank Japan Project: Study Design and Profile*, 27 J Epidemiol. S2–S8 (2017).

55 Japan Agency for Medical Research and Development, *Biobank-Construction and Utilization Biobank for Genomic Medicine Realization (Platform Program for Promotion of Genome Medicine*), https://www.amed.go.jp/en/program/list/14/05/008.html (accessed Mar. 5, 2026).

56 Japan Accreditation Board (JAB). Biobanking (JIS Q 20387). (jab.or.jp/en/service/biobanking) (accessed Mar. 5, 2026).

57 Regulation (EU) 2016/679 (GDPR).

58 Winickoff & Winickoff, *supra* note 9.

59 Miller & Gold, *supra* note 16.

60 Virani & Longstaff, *supra* note 25.

61 Miller & Gold, *supra* note 16.

62 Winickoff, *supra* note 11.

63 Garden, Hawkins & Winickoff, *supra* note 12.

64 M. Vaz, M. Vaz & K. Srinivasan, *The Views of Ethics Committee Members and Medical Researchers on the Return of Individual Research Results and Incidental Findings, Ownership Issues and Benefit Sharing in Biobanking Research in a South Indian City*, 18 Dev  World  Bioeth. 321–330 (2018).

65 Winickoff & Winickoff, *supra* note 9.

66 Langhof, Schwietering & Strech, *supra* note 3.

67 International Organization for Standardization, *supra* note 7.

68 ISBER, *supra* note 8.

69 Winickoff, *supra* note 11.

70 Miller & Gold, *supra* note 16.

71 Garden, Hawkins & Winickoff, *supra* note 12.

72 J. Vaught et al., *Biobankonomics: Developing a Sustainable Business Model Approach for the Formation of a Human Tissue Biobank*, 2011 J Natl  Cancer  Inst  Monogr. 24–31 (2011).

73 International Organization for Standardization, *supra* note 7.

74 ISBER, *supra* note 8.

75 Miller & Gold, *supra* note 16.

76 Smith, *supra* note 24.

77 Jordan, Liddicoat & Liddell, *supra* note 43.

78 Simon & Robienski, *supra* note 44.

79 C. Staunton & J. de Vries, *The Governance of Genomic Biobank Research in Africa: Reframing the Regulatory Tilt*, 7 J Law  Biosci. lsz018 (2020), https://doi.org/10.1093/jlb/lsz018.

80 A. Yakubu et al., *Model Framework for Governance of Genomic Research and Biobanking in Africa – A Content Description*, 1 AAS Open  Res. 13 (2018), https://doi.org/10.12688/aasopenres.12844.2.

81 J. Aramoana & J. Koea, *An Integrative Review of the Barriers to Indigenous Peoples Participation in Biobanking and Genomic Research*, 6 JCO Glob  Oncol. 83–91 (2020).

82 Japan Accreditation Board (JAB), *supra* note 56.

83 T. Takai-Igarashi et al., *Security Controls in an Integrated Biobank to Protect Privacy in Data Sharing: Rationale and Study Design*, 17 BMC Med  Inform  Decis  Mak. 100 (2017).

84 Jordan, Liddicoat & Liddell, *supra* note 43.

85 Simon & Robienski, *supra* note 44.

86 Goebel et al., *supra* note 4.

87 Winickoff & Winickoff, *supra* note 9.

88
Johnsson, *supra* note 5.

89 Garden, Hawkins & Winickoff, *supra* note 12.

90 International Organization for Standardization, *supra* note 7.

91 ISBER, *supra* note 8.

92 Winickoff & Winickoff, *supra* note 9.

93 Miller & Gold, *supra* note 16.

94 Virani & Longstaff, *supra* note 25.

95 International Organization for Standardization, *supra* note 7.

96 ISBER, *supra* note 8.

97 Garden, Hawkins & Winickoff, *supra* note 12.

98 Smith, *supra* note 24.

99 Virani & Longstaff, *supra* note 25.

100 Watson et al., *supra* note 26.

101 Porteri, Togni & Pasqualetti, *supra* note 52.

102 Jordan, Liddicoat & Liddell, *supra* note 43.

103 Simon & Robienski, *supra* note 44.

104 Goebel et al., *supra* note 4.

105
Johnsson, *supra* note 5.

106 Garden, Hawkins & Winickoff, *supra* note 12.

